# Effect of Psyllium Husk, Bran, and Raw Wheat Germ Addition on the Rheological Characteristics of Arabic (Pita) Bread Dough

**DOI:** 10.1155/2020/8867402

**Published:** 2020-12-30

**Authors:** Ahmad Aldughpassi, Tasleem Zafar, Jiwan S. Sidhu, Fatima Al-Hassawi, Mohammad Mirza Abdullah, Amani Al-Othman

**Affiliations:** ^1^Department of Food Science & Nutrition, College of Life Sciences, Kuwait University, P.O. Box. 5969, Safat, 13060, Kuwait; ^2^Food Science & Nutrition Program, P.O. Box. 24885, Safat, 13109, Kuwait; ^3^Information and Communications Technology Dept, Kuwait Institute for Scientific Research, P.O. Box. 24885, Safat, 13109, Kuwait

## Abstract

Arabic bread (*khubuz*) made from white flour is the staple food in the Arabic countries but has now become popular all over the world. A different approach of producing high fiber bread with improved quality can be produced using white flour with added mill fractions, but the addition of mill fractions has been shown to adversely affect the dough characteristics. Therefore, the effect of adding mill fractions on the rheological characteristics of dough was investigated using Brabender Farinograph and Extensograph with the major objective of eliminating their deleterious effects on dough quality, mainly by using psyllium husk, and also reported as an excellent source of soluble dietary fiber. Addition of fine bran, coarse bran, and raw wheat germ decreased the extensibility and resistance to extension and area under curve, lower dough stability, but enhanced water absorption and peak time. Addition of psyllium husk, though reduced the extensibility, but did not affect the area under the curve adversely, thus overcame some of the negative effects on rheological characteristics of the white flour dough. It was concluded that the use of psyllium husk will evidently help the bakers to produce nutritious and acceptable quality Arabic bread.

## 1. Introduction

Most of the nations have now established grains as the base of healthy eating pattern and are recommending multiple servings of whole grains [1]. Moreover, whole grains are rich source of many antioxidants which protect us against some of the chronic diseases, including cardiovascular diseases, diabetes, and many types of cancer [2]. Whole wheat flour and whole wheat bread are reported to lower cholesterol in rats [3], apart from providing a wide range of nutrients and biologically active compounds such as dietary fiber, B and E vitamins, selenium, zinc, copper, magnesium, and phenolic compounds, which work synergistically to reduce the incidence of various diseases [4]. Bile binding by wheat bran may contribute to cancer prevention and other healthful properties [5].

The Kuwaiti population mainly consumes white Arabic bread (*khubuz*), white toast bread, and highly polished rice; this evidently results in lower intake of dietary fiber. The incidence of constipation and its related diseases like diverticular, appendicitis, piles, hemorrhoids, and anal fissures is significantly high among children, adults, and the elderly in the Kuwaiti population. This necessitates the need for commonly consumed food products such as Arabic bread enriched with both the soluble and insoluble dietary fiber sources. According to the latest figures available [6], a total of 24,300 inpatients (diabetics, and cardiac, hypertension and cancer cases) had to be served with daily meals having high fiber contents in the various clinics of the country. The catering companies would have to produce nearly 16,000 loaves of high fiber Arabic bread daily to meet these requirements. In addition, many thousands of outpatients visiting weight clinics would like to consume such high-fiber baked products.

The consumption of toast bread is steadily increasing at about 10% per annum, whereas Arabic bread consumption has almost stagnated. More than 90% of the bread being consumed in Kuwait is made from white flour, which is depleted of natural dietary fiber. The total dietary fiber content of whole wheat flour is 10.2% compared with 2.5% for white flour. On the other hand, the values for total dietary fiber in wheat bran range between 40 and 44%, thus making it an ideal natural supplement for producing high-fiber baked products [7]. The Kuwaiti consumers prefer white flour Arabic bread over that of whole wheat flour bread, mainly because of its lighter color and superior eating quality. Use of psyllium is expected not to affect the crust color of these baked products adversely, thus retaining their consumer acceptability.

The satiety value of whole meal bread has been reported to be significantly higher than white bread [8]. Thus, one would eat more white bread leading to higher calorie intake, as white bread has a greater energy density (2.3 cal./g) than whole meal bread (2.16 cal./g). In general, subjects consuming whole meal or high-fiber breads feel fuller than those consuming white bread, as the former has higher satiety value. Wheat bran and germ, because of their flavor, and a good amount of proteins of high biological value, have also been reported to be a rich source of B-complex vitamins and minerals [9]. The dough rheological characteristics are good predictors of the quality of the finished products (Arabic bread), but the addition of coarse bran, fine bran, and raw wheat germ has been reported to adversely affect the rheological performance of wheat dough [7]. The major objective of this investigation was to optimize the use of psyllium husk to eliminate the adverse effects of addition of mill fractions on the rheological characteristics of wheat flour dough required for Arabic bread making.

## 2. Materials and Methods

### 2.1. Raw Materials

Whole wheat flour (WWF), white flour (WF), coarse bran (CB), fine bran (FB), and wheat germ (WG) samples were obtained from the Kuwait Flour Mills & Bakeries Co., Shuwaikh. Psyllium husk was procured from India through a local importer. These samples were analyzed for moisture (method 44-19), crude protein (method 46-12), crude fat (method 30-25), and ash (method 08-01) contents, according to standard AACC methods [10]. Coarse bran fraction comes from the outer layers of wheat grain and is obtained from the break roll section, whereas the fine bran (also called “shorts” in USA) which comes from the inner bran layers of wheat grain closer to aleurone and also includes the aleurone layer is obtained from reduction roll section of a roller flour mill.

### 2.2. Rheological Characteristics

#### 2.2.1. Dough Making for Rheological Studies

For farinograph studies, as for the constant flour methods, 50 g of flour (14% moisture basis) was used. The temperature of water was adjusted to 30 ± 0.2°C. The water bath (30 ± 0.2°C) was turned on at least one hour before using this instrument. After adding flour, the instrument was turned on for 1 min before the water was added from the burette into the right front corner of the bowl, continued mixing till the center of the graph arrived at the 500 BU line. The amount of water needed to reach this height of 500 BU was taken as farinograph water absorption (FWA).

For extensograph studies, 300 g of flour (14% moisture basis) and 6 g salt (dissolved in water) were used in a large 300 g bowl on the farinograph instrument. Using 2% less water than the FWA to compensate for the use of salt, dough was mixed to arrive at the peak height of dough formation. At the end of mixing, 150 ± 0.1 g of dough was scaled, gave 20 revolutions in the extensograph rounder unit, and then carefully shaped into a cylinder on the shaping unit. This cylinder was loaded into the lightly greased dough holder, clamped with the holder pins. Three cylinders so obtained were stored in the humidity chamber of the extensograph till testing was done for extensibility and resistance to extension at 45, 90, and 135 min of resting. After each stretching, dough was made into a cylinder the same way as explained above.

The replacement levels of white flour with coarse bran, fine bran, wheat germ, and psyllium husk were finalized after a number of preliminary studies in Hobart dough mixer for obtaining optimum dough with subjectively feeling of the dough quality. Levels higher than this resulted in detrimental dough quality with a very weak structure that was found not suitable for good baking performance.

#### 2.2.2. Brabender Farinograph

Farinograph tests for wheat flour and various optimized blends containing raw wheat germ, bran fractions, and psyllium were conducted using a Brabender Farinograph (CW Brabender Co., Germany) equipped with a 50 g stainless-steel bowl. The constant flour weight procedure was used for obtaining farinograms, as per the AACC Method 54-21 [10]. The volume of water required to produce a curve with a maximum resistance centered on the 500-BU line was recorded as the farinograph water absorption (FWA). Brabender Farinograph (Brabender, Germany) was used to measure these properties of wheat flour dough (dough development time, peak time, tolerance index, stability, time to breakdown, valorimeter value).

#### 2.2.3. Brabender Extensograph

Extensograph tests for wheat flour and various optimized blends containing raw wheat germ, bran fractions, and psyllium were conducted using a Brabender Extensograph. Dough elasticity and extensibility assessed by Brabender Extensograph (Brabender, Germany) were used to obtain three measurements (resistance to extension, extensibility, area under the curve) as per the AACC Method 54-10 [10].

### 2.3. Statistical Analysis

All the chemical analyses are reported on moisture free basis. All the experimental data obtained were analyzed statistically for analysis of variance, for statistical significance using Duncan's New Multiple Range Test (SAS Program Windows Version 6.08), and inference reported at the appropriate places. Significance was accepted at the *P* = 0.05 level. For most of the results, the mean values with standard deviations are reported in the tables.

The reagents used in the chemical analyses of this work were of analytical grade.

## 3. Results and Discussion

### 3.1. Chemical Composition

**Figure 1:**
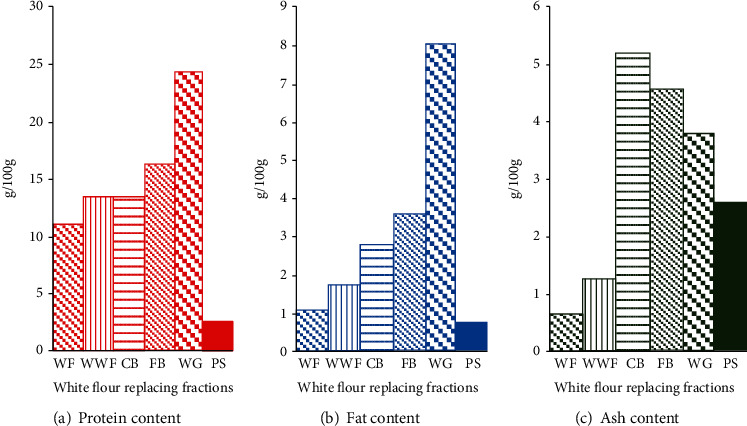
Protein, fat, and ash content of various fractions of wheat compared with the psyllium husk. Legend: WF: white flour; WWF: whole wheat flour; CB: coarse bran; FB: fine bran; WG: wheat germ; PS: psyllium husk.

Whole wheat flour, white flour, coarse bran, fine bran, psyllium, and germ used in this study were analyzed for crude protein, crude lipids, and ash contents. The proximate analyses (% dry basis) for WF, WWF, CB, FB, WG, and PS have been presented in Figure 1. The ash, protein, and fat contents of flour, bran, and germ were closer to those reported in the literature [11]. Bran fractions and raw wheat germ were found to be rich in minerals and protein. As expected, the ash content was the lowest in white flour (0.64%) but was higher in bran and germ fractions. The protein contents of raw wheat germ were the highest (24.24%) as compared with psyllium, which had the lowest value (2.53%). The wheat germ, as expected, was especially richer in fat (7.99%) contents. The higher fat and protein contents of fine bran samples compared with coarse bran may be due to the inclusion of layer and the germ, because most of these two components end up in these fine bran aleurone mill fractions during the normal wheat milling process. Unfortunately, during white flour production, coarse and fine bran as well as the germ are separated into mill fractions which constitute what is called as mill feed, and it goes into cattle feed manufacture.

### 3.2. Farinograph Characteristics

WF, WWF, and various combinations with coarse bran, fine bran, and psyllium were evaluated for dough rheology using a Brabender Farinograph and Brabender Extensograph, and the results are presented in Tables 1–12. Farinograph is a commonly used instrument in the laboratory for obtaining correct water absorption values for reaching the required dough consistency for producing good quality baked goods. FWA values were higher for WWF than for WF (Table 1). FWA increased progressively with increases in the addition of coarse bran as well as fine bran fractions to the WF. With the addition of fine bran as well as course bran, FWA values increased significantly at 20% level (Table 1). At 10% addition of these bran factions, the additional water picked up got neutralized by the dilution of the gluten content in WF. Regardless of the level of bran addition, it is known to adversely affect the formation of the proper structure during mixing of dough [12].

As the bran particles have finer size, their surface area increases leading to higher water absorption. Similar findings have also been reported by Pauly et al. [13]. The FWA for WF in our study is almost identical to the one reported by Hemery et al. [14] but the FWA for WWF in our study is slightly lower than the one reported by them. This may possibly be due to the differences in the quality and quantity of gluten proteins or the particle size of flour or bran particles. The ultrafine milling of flour to 18.36 to 57.96 *μ*m particle size has been reported to result in higher Farinograph water absorption from 59.1 to 72.9% [15]. Another important factor that affects the FWA is the amount of the damaged starch content of a normally milled flour [16]. The grinding of starch granules to finer size increased the surface area, thus leading to higher water absorption values [17]. The addition of fine bran and coarse bran at 20% level significantly increased the FWA, peak time, dough stability, and valorimeter value (Table 1). The bran dietary fiber has a large numbers of hydroxyl groups which can bind tremendous amount water due to hydrogen bonding, much more than the major polymers present in wheat dough, i.e., gluten and starch [18–20]. The wheat germ being a rich source of glutathione, a known reducing agent, made the dough weaker during mixing; thus, the water absorption had to be reduced by nearly 4% than the WF to get an optimum dough consistency of 500 BU (Table 1).

Every 1% of psyllium added resulted in an approximately 4% increase in the FWA value (Table 2). As expected, the peak time was higher (8 min) with WF than with WWF (6 min). Addition of higher amounts of bran or germ fractions lowered the peak time further. In contrast, addition of psyllium increased the peak time significantly (11 min). Dough stability values were higher for WF (16.5 min) than for WWF (9 min). Fine bran addition to WF significantly decreased the dough's stability, whereas the addition of coarse bran had no effect on this parameter. Wheat germ addition drastically reduced the dough's stability (3.5 min) because of the presence of reducing substances such as glutathione [9], which was also reflected in higher MTI values. Marti et al. [21] have investigated the weakening of dough containing wheat germ and have shown glutathione to be the major component responsible for the deterioration of rheological characteristics of dough. The reason for lower dough stability in WWF than in WF could be due to the activity of various proteolytic enzymes present in the aleurone layer which is a normal constituent of WWF. The same could be true when fine bran fraction is added to WF [9]. As dough stability and MTI values are inversely related, lower MTI values are desirable in Arabic bread making. Interestingly, the addition of psyllium at all levels of addition increased dough stability (11 min). In terms of dough stability, MTI, and valorimeter values, various combinations of WF, coarse bran ,and psyllium produced dough characteristics which were quite desirable for Arabic bread making.

Psyllium fiber has recently been shown to influence the starch hydration properties, pasting behavior, and dough rheology even under cold conditions [22]. Highly fibrillated cellulose has a large surface area, and these smaller fibrils have significantly higher interaction with psyllium heteroxylan resulting in a compact structure [23]. Psyllium husk, a rich source of soluble fiber fraction made of heteroxylan, has been shown to possess remarkable water absorption capacity and gelling properties [24]. According to them, the main functional component (F60) of psyllium husk is a complex branched heteroxylan structure that possesses unique rheological properties and offers tremendous functional applications in food industry. Many of the effects on the psyllium addition to WF on the rheological properties can be explained due to these interactions between heteroxylans with the gluten proteins.

The addition of psyllium to WF at various levels (from 1 to 5%) significantly increased the water absorption (FWA) and peak time but decreased the dough stability and valorimeter values (Table 2). The heteroxylans in psyllium husk have a tremendous amount of hydroxyl groups which have higher water binding capacity [24]. As expected, in the presence of 10% fine bran (FB), but with an increasing levels of psyllium husk addition, both the FWA and peak time increased significantly (Table 3). In the presence of fine bran and psyllium competing for available water in the system, the dough proteins obviously took longer time to develop viscoelastic structure. However, with higher level of 20% fine bran and increasing amounts of psyllium addition, both the FWA and peak time increased significantly (Table 4), but the dough stability increased significantly only at higher levels of psyllium (4 and 5%). Similarly, the valorimeter values were significantly higher at 4 and 5% level of psyllium than the lower levels of 1 to 3% of psyllium [25, 26]. Extractable phenolics and proteins obtained from wheat bran have been shown to give positive correlation with dough strength and dough development time by Navrotskyi et al. [27].

When coarse bran (CB) was added at 10% level along with the increasing amounts of psyllium (from 1 to 5%), both the FWA and peak time increased significantly (Table 5), but the dough stability decreased at all levels of psyllium addition with no change in valorimeter values. Interestingly, at higher levels of CB (20%) in the presence of increasing levels of psyllium (1 to 5%), the FWA, dough stability, peak time, and valorimeter values were significantly increased (Table 6). The higher dough stability and valorimeter values at higher levels of psyllium and bran could be explained due to the interaction of ferulic acid present in wheat bran pentosans that strengthens the gluten proteins of wheat flour as suggested by various workers [28–30]. The arabinoxylan (AX) in wheat bran is also known to strengthen the AX gels which increase their ability to bind higher amounts of water during dough making [31–33]. Recently, Parenti et al. [12] have also shown the bran and germ to have a negative effect on the rheological performance of the dough.

### 3.3. Extensograph Characteristics

The extensibility (E), resistance to extension (R-to-E) and area (A) under the extensigram of dough were significantly higher in WF than the WWF (Table 7). Compared to control WF, the addition of fine bran both at 10 and 20% level significantly reduced the E, R-to-E, and A values for dough; however, the coarse bran addition at both the 10 and 20% level significantly decreased the extensibility, but increased the R-to-E and A values. The addition of wheat germ at both levels (because of its higher glutathione content) significantly increased E values and reduced R-to-E values but slightly increased the A values when compared with the WF control sample (Table 7). Psyllium addition at varying levels from 1 to 5% to the WF decreased E values (from 25.9 to 14.1 cm) but increased R-to-E values (from 11.9 to 17.1 cm) and increased area (A) under the graph (from 213 to 229.6 cm^2^), thus indicating greater dough stability (Table 8). When psyllium husk was added along with the 10% levels of fine bran, significantly decreased the E values from 25.9 to 11.2 cm, R-to-E values from 11.9 to 10.9 cm, and A under the graph from 213 to 83 cm^2^, indicating a slight weakening of the dough (Table 9). Higher level of fine bran (20%) addition along with psyllium husk further weakened the dough structure as shown by the decrease in E and A values, but no significant change in R-to-E values (Table 10). In case of addition of coarse bran (10 and 20% levels) along with psyllium husk at all levels from 1 to 5%, the influence on the extensibility, resistance to extension, and area under the curve decreased significantly (Tables 11 and 12). Coarse bran fractions originate from the outermost layers of wheat kernel during the roller flour milling process. These outer layers are known to contain small epicarp hairy structure on their surfaces which have a strong weakening influence on the gluten structure and can even lead to lower specific volume of the baked bread [34]. The increased amounts of arabinoxylans and fiber contents have been shown to decrease extensibility and increase the R-to-E. The higher amounts of bran fractions also increase the total protein content but decrease the levels of gluten concentrations [35, 36], and the bran addition also reduces the gluten polymerization, resulting in the breakdown of the gluten structure [34].

The addition of bran to flour has been shown to affect the rheological characteristics due to many reasons. The fiber constituents present in bran and psyllium compete with the available water during dough missing, with other major polymers, such as gluten and starch, thus hindering the strong gluten network, leading to lower resistance to extension, but weaker gluten with more extensibility [37–39]. Secondly, the ferulic acid present in water soluble pentosans has been shown to strengthen the gluten network [31, 32]. Most of the phenolic compounds are concentrated mainly in the outer layers of cereal grains which constitute bran [40]. The physical interaction of bran components with the gluten network is the major reason of the negative effect of fiber addition on the decreased extensibility values and higher R-to-E values [38]. Ahluwalia et al. [41] have studied the effect of psyllium addition on the rheological properties of wheat flour. With the addition of psyllium, they observed higher water absorption and stability but decreased mixing tolerance. Rao and Rao [42] have studied the effect of incorporating wheat bran on the rheological characteristics and bread-making quality of flour. They used higher levels than our study and found that a maximum of 30% wheat bran could be added to flour to obtain an acceptable bread. At the higher level of dietary fiber (bran, psyllium) addition, gluten proteins of the WF can partially manage the negative effects of fiber addition on the dilution of gluten proteins [35, 36, 39, 43]. Similar trends in dough rheological characteristics were observed with various combinations of coarse bran and psyllium addition to the WF during this study or with chickpea flour addition in earlier studies [44, 45].

## 4. Conclusions and Recommendations

The results presented here in this paper bring out clearly the influence of adding coarse bran, fine bran, wheat germ, and psyllium husk on various rheological characteristics as measured by Farinograph and Extensograph. The major reasons for the weakening of the gluten structure are the competition for water between starch, gluten, and fiber constituents that is available in the dough system. This study has pointed out that the addition of psyllium husk, though reduced the extensibility, but did not affect the area under the curve adversely. The use of psyllium husk has, thus, overcome some of the negative effects of wheat germ and bran fractions on the rheological characteristics of the white flour dough. It was concluded that the use of psyllium husk will evidently help the bakers to produce nutritious and acceptable quality Arabic bread. The various phenolic compounds present in the bran layers need to be further investigated if they play any significant role in strengthening the gluten proteins during the dough mixing process. More research is also needed to suggest ways to eliminate or reduce the negative effects of wheat bran, wheat germ, and psyllium husk addition on dough rheology. Addition of these fiber sources is very important for the baking industry to produce bakery products rich in dietary fiber, vitamins, minerals, and antioxidant phytochemicals to provide health benefits to the consumers against many noncommunicable diseases, such as obesity, type 2 diabetes, cardiovascular diseases, and many types of cancers.

## Figures and Tables

**Table 1:** Farinograph characteristics of dough made from flour, bran fractions, and germ blends.

Sample code	FWA %	Peak time (min)	Dough stability (min)	MTI (BU)	Valorimeter value
WWF	68.5^a^	6.0^a^	9.0^a^	60^a^	80^a^
WF	64.5^b^	8.0^b^	16.5^b^	25^b^	95^b^
WF+10% fine bran	64.0^b^	7.0^b^	9.0^c^	40^c^	81^c^
WF+20% fine bran	67.0^c^	5.5^c^	8.0^c^	60^d^	79^c^
WF+10% coarse bran	64.0^b^	7.0^b^	16.0^b^	5^b^	95^b^
WF+20% coarse bran	67.0^c^	6.0^a^	17.0^b^	15^b^	95^b^
WF+10% wheat germ	61.0^d^	4.0^d^	3.5^d^	135^e^	60^d^
WF+20% wheat germ	60.0^d^	4.5^d^	3.5^d^	205^f^	58^d^

FWA: Farinograph water absorption; BU: Brabender units; MTI: mixing tolerance index: WWF: whole wheat flour; WF: white flour. Mean values (*N* = 3) with same superscripts for any parameter do not differ significantly in a column (*P* = 0.05).

**Table 2:** Farinograph characteristics of dough made from flour and psyllium blends.

Sample code	FWA %	Peak time (min)	Dough stability (min)	MTI (BU)	Valorimeter value
WF	64.5^a^	8.0^a^	16.5^a^	25^a^	95^a^
WF+ 1% psyllium	67.0^b^	11.5^b^	13.5^b^	10^a^	94^a^
WF+ 2% psyllium	71.5^c^	11.0^b^	14.0 ^b^	25^a^	94^a^
WF+ 3% psyllium	76.0^d^	11.0^b^	13.0 ^b^	10^a^	94^a^
WF+ 4% psyllium	80.0^e^	11.0^b^	11.0^b^	30^a^	94^a^
WF+ 5% psyllium	84.0^f^	11.0^b^	12.0^b^	20^a^	94^a^

FWA: Farinograph water absorption; BU: Brabender units; MTI: mixing tolerance index; WF: white flour. Mean values (*N* = 3) with same superscripts for any parameter do not differ significantly in a column (*P* = 0.05).

**Table 3:** Farinograph characteristics of dough made from flour, fine bran, and psyllium blends.

Sample code	FWA %	Peak time (min)	Dough stability (min)	MTI (BU)	Valorimeter value
WF	64.5^a^	8.0^a^	16.5^a^	25^a^	95^a^
WF+10% fine bran+1% psyllium	67.5^b^	6.5^b^	10.5^b^	45^a^	87^b^
WF+10% fine bran +2% psyllium	72.0^c^	9.0^ac^	10.0^b^	55^a^	87^b^
WF+10% fine bran +3% psyllium	81.0^d^	9.0^ac^	11.0^b^	45^a^	87^b^
WF+10% fine bran +4% psyllium	86.0^e^	10.0^c^	12.0^a^	40^a^	92^a^
WF+10% fine bran +5% psyllium	93.5^f^	10.0^c^	13.5^a^	25^a^	94^a^

FWA: Farinograph water absorption; BU: Brabender units; MTI: mixing tolerance index; WF: white flour. Mean values (*N* = 3) with same superscripts for any parameter do not differ significantly in a column (*P* = 0.05).

**Table 4:** Farinograph characteristics of dough made from flour, fine bran, and psyllium blends.

Sample code	FWA %	Peak time (min)	Dough stability (min)	MTI (BU)	Valorimeter value
WF	64.5^a^	8.0^a^	16.5^a^	25^a^	95^a^
WF+20% fine bran +1% psyllium	70.0^b^	6.0^b^	9.5^b^	75^b^	84^b^
WF+20% fine bran +2% psyllium	76.0^c^	7.0^c^	9.0^b^	70^b^	85^b^
WF+20% fine bran +3% psyllium	84.0^d^	8.5^a^	9.0^b^	70^b^	85^b^
WF+20% fine bran +4% psyllium	89.0^e^	9.5^d^	10.0^c^	55^c^	91^a^
WF+20% fine bran +5% psyllium	92.0^f^	10.5^e^	12.0^d^	45^c^	93^a^

FWA: Farinograph water absorption; BU: Brabender units; MTI: mixing tolerance index; WF: white flour. Mean values (*N* = 3) with same superscripts for any parameter do not differ significantly in a column (*P* = 0.05).

**Table 5:** Farinograph characteristics of dough made from flour, coarse bran, and psyllium blends.

Sample code	FWA %	Peak time (min)	Dough stability (min)	MTI (BU)	Valorimeter value
WF	64.5^a^	8.0^a^	16.5^a^	25^a^	95^a^
WF+10% coarse bran+1% psyllium	75.0^b^	10.5^b^	13.0^b^	30^b^	94^a^
WF+10% coarse bran +2% psyllium	82.0^c^	10.0^b^	12.0^b^	30^b^	94^a^
WF+10% coarse bran +3% psyllium	90.0^d^	13.0^c^	15.0^b^	20^a^	94^a^
WF+10% coarse bran +4% psyllium	95.0^e^	12.5^c^	11.5^b^	30^b^	94^a^
WF+10% coarse bran +5% psyllium	100.0^f^	13.5^c^	10.5^b^	35^b^	94^a^

FWA: Farinograph water absorption; BU: Brabender units; MTI: mixing tolerance index; WF: white flour. Mean values (*N* = 3) with same superscripts for any parameter do not differ significantly in a column (*P* = 0.05).

**Table 6:** Farinograph characteristics of dough made from flour, coarse bran, and psyllium blends.

Sample code	FWA %	Peak time (min)	Dough stability (min)	MTI (BU)	Valorimeter value
WF	64.5^a^	8.0^a^	16.5^a^	25^a^	95^a^
WF+20% coarse bran +1% psyllium	78.0^b^	8.0^a^	14.0^a^	5^b^	94^a^
WF+20% coarse bran +2% psyllium	84.0^c^	9.5^b^	13.0^a^	5^b^	94^a^
WF+20% coarse bran +3% psyllium	89.0^d^	10.0^b^	13.0^a^	10^b^	94^a^
WF+20% coarse bran +4% psyllium	94.0^e^	10.0^b^	13.0^a^	10^b^	94^a^
WF+20% coarse bran +5% psyllium	99.0^f^	13.5^c^	13.0^a^	5^b^	94^a^

FWA: Farinograph water absorption; BU: Brabender units; MTI: mixing tolerance index; WF: white flour. Mean values (*N* = 3) with same superscripts for any parameter do not differ significantly in a column (*P* = 0.05).

**Table 7:** Extensograph characteristics of dough made from flour, germ, bran fractions, and psyllium blends.

Sample code	Extensibility (cm)			Resistance to extension (cm)			Area under the curve (cm^2^)		
	45	90	135	45	90	135	45	90	135
Whole wheat flour (WWF)	16.7^a^_f_	14.9_fg_	13.3_g_	7.2^a^_f_	9.5_g_	11.3_h_	83.5^a^_f_	100.5_g_	104.9_g_
White flour (WF)	25.9^b^_f_	24.4_g_	23.5_fg_	11.9^b^_f_	15.2_g_	15.4_g_	213.0^b^_f_	254.6_g_	247.7_g_
WF+10% fine bran	19.2^c^_f_	18.4 _f_	17.5_g_	10.8^bc^_f_	13.4_g_	15.3_h_	150.3^c^_j_	177.6_k_	185.4_k_
WF+20% fine bran	14.9^d^_f_	14.1_f_	14.1_f_	10.1^c^_f_	12.8_g_	13.2_g_	119.7^d^_j_	133.5_jk_	137.2_k_
WF+10% coarse bran	16.6^e^_f_	15.0_f_	15.3_f_	14.2^d^_f_	17.8_g_	18.6 _g_	174.4^e^_j_	191.4_jk_	202.9_k_
WF+20% coarse bran	11.9^m^_f_	11.2_f_	9.7_g_	13.1^bd^_f_	16.4_g_	18.6_h_	130.6^f^_j_	134.8_k_	127.5_jk_
WF+10% wheat germ	24.6^b^_f_	21.4_g_	20.2_h_	3.9^e^_g_	4.0_g_	4.6_g_	175.5^g^_j_	177.1_j_	182.3_j_
WF+20% wheat germ	22.7^n^_f_	21.3_f_	20.8_f_	4.0^e^_g_	4.2_gh_	4.7_h_	179.5^g^_j_	182.5_j_	185.2_j_

Mean values (*N* = 3) with same superscripts for any parameter do not differ significantly in a column (*P* = 0.05). Mean values with same subscripts for any parameter (i.e., extensibility, resistance to extension, or area under the curve) at 45, 90, and 135 minutes do not differ significantly in a row (*P* = 0.05).

**Table 8:** Extensograph characteristics of dough made from flour and psyllium blends.

Sample code	Extensibility (cm)			Resistance to extension (cm)			Area under the curve (cm^2^)		
	45	90	135	45	90	135	45	90	135
White flour (WF)	25.9^a^_f_	24.4_g_	23.5_g_	11.9^a^_f_	15.2_g_	15.4_g_	213.0^a^_f_	254.6_g_	247.7_g_
WF+ 1% psyllium	18.1^b^_f_	17.4_f_	17.6_f_	13.8^b^_f_	18.2_g_	18.2_g_	175.7^a^_f_	227.8_g_	241.1_g_
WF+ 2% psyllium	16.6^b^_f_	15.9_f_	15.6_f_	15.6^b^_f_	18.1_g_	18.3_g_	185.4^ab^_f_	215.1	211.3_g_
WF+ 3% psyllium	16.3^b^_f_	17.4_f_	16.0_f_	13.8^b^_f_	18.2_g_	18.2_g_	180.6^ab^_f_	241.0_g_	224.9_g_
WF+ 4% psyllium	12.6^c^_f_	9.8_g_	8.8_g_	16.7^b^_f_	18.2_g_	18.2_g_	229.6^a^_f_	234.6 _f_	256.3_f_
WF+ 5% psyllium	14.1^d^_f_	13.4_f_	10.2_f_	17.1^b^_f_	18.2_f_	18.3_f_	188.8^b^_f_	196.6 _f_	177.6_f_

Mean values (*N* = 3) with same superscripts for any parameter do not differ significantly in a column (*P* = 0.05). Mean values with same subscripts for any parameter (i.e., extensibility, resistance to extension, or area under the curve) at 45, 90, and 135 minutes do not differ significantly in a row (*P* = 0.05).

**Table 9:** Extensograph characteristics of dough made from flour, fine bran, and psyllium blends.

Sample code	Extensibility (cm)			Resistance to extension (cm)			Area under the curve (cm^2^)		
	45	90	135	45	90	135	45	90	135
White flour (WF)	25.9^a^_f_	24.4_g_	23.5_g_	11.9^a^_f_	15.2_g_	15.4_g_	213.0^a^_f_	254.6 _g_	247.7 _g_
WF+10% fine bran +1% psyllium	17.6^b^_f_	16.4_g_	16.6_g_	9.7^b^_f_	12.2_g_	13.3_h_	137.9^b^_f_	156.2 _g_	171.3 _g_
WF+10% fine bran +2% psyllium	15.1^cd^_f_	14.0_f_	13.5_f_	13.5^a^_f_	16.0_g_	18.0_h_	149.4^b^_f_	164.2 _fg_	169.9 _g_
WF+10% fine bran +3% psyllium	15.7^c^_f_	14.9_f_	14.0_f_	10.5^ab^_f_	14.0_g_	15.7_h_	130.2^b^_f_	157.6_g_	162.1_g_
WF+10% fine bran +4% psyllium	14.7^d^_f_	13.0_f_	12.7_f_	10.9^a^_f_	16.0_g_	18.2_h_	125.3^b^_f_	149.1_g_	167.0_g_
WF+10% fine bran +5% psyllium	12.6^e^_f_	12.0_f_	11.9_f_	12.7^a^_f_	16.5_g_	18.2_h_	127.1^b^_f_	150.0 _g_	159.7_g_

WWF: whole wheat flour; WF: white flour. Mean values (*N* = 3) with same superscripts for any parameter do not differ significantly in a column (*P* = 0.05). Mean values with same subscripts for any parameter (i.e., extensibility, resistance to extension, or area under the curve) at 45, 90, and 135 minutes do not differ significantly in a row (*P* = 0.05).

**Table 10:** Extensograph characteristics of dough made from flour, fine bran, and psyllium blends.

Sample code	Extensibility (cm)			Resistance to extension (cm)			Area under the curve (cm^2^)		
	45	90	135	45	90	135	45	90	135
White flour (WF)	25.9^a^_f_	24.4_g_	23.5_g_	11.9^a^_f_	15.2_g_	15.4_g_	213.0^a^_f_	254.6_g_	247.7_g_
WF+20% fine bran+1% psyllium	13.7^b^_f_	13.6_f_	13.3_f_	10.0^a^_f_	12.5_g_	14.4_h_	107.5^b^_f_	134.2_g_	144.2_g_
WF+20% fine bran+2% psyllium	13.5^b^_f_	13.1_fg_	12.1_g_	9.4^a^_f_	12.6_g_	15.0_h_	102.7^bc^_f_	126.0_g_	138.1_g_
WF+20% fine bran+3% psyllium	11.4^c^_f_	11.2_f_	10.6_f_	11.3^a^_f_	15.1_g_	17.5_h_	101.6^bc^_f_	124.9_g_	131.7_g_
WF+20% fine bran+4% psyllium	12.3^c^_f_	11.7_f_	11.0_f_	10.1^a^_f_	12.8_g_	16.1_h_	94.1^cd^_f_	110.4_g_	126.1_g_
WF+20% fine bran+5% psyllium	11.2^d^_f_	9.3_g_	8.9_g_	10.9^a^_f_	14.7_g_	16.2_h_	83.0^d^_f_	103.8_g_	98.7_g_

Mean values (*N* = 3) with same superscripts for any parameter do not differ significantly in a column (*P* = 0.05). Mean values with same subscripts for any parameter (i.e., extensibility, resistance to extension, or area under the curve) at 45, 90, and 135 minutes do not differ significantly in a row (*P* = 0.05).

**Table 11:** Extensograph characteristics of dough made from flour, coarse bran, and psyllium blends.

Sample code	Extensibility (cm)			Resistance to extension (cm)			Area under the curve (cm^2^)		
	45	90	135	45	90	135	45	90	135
White flour (WF)	25.9^a^_f_	24.4_g_	23.5_g_	11.9^ab^_f_	15.2 _g_	15.4_g_	213.0^a^_f_	254.6_g_	247.7_g_
WF+10% coarse bran+1%psyllium	17.1^b^_f_	16.2_f_	14.7_g_	11.3^ab^_f_	15.9 _g_	18.5_h_	142.8^b^_f_	177.7_g_	190.2_g_
WF+10% coarse bran+2%psyllium	17.4^b^_f_	15.9_g_	13.8_h_	9.3^a^_f_	13.9 _g_	16.9_h_	121.2^c^_f_	158.9_g_	154.4_g_
WF+10% coarse bran+3%psyllium	16.2^c^_f_	13.6_g_	12.4_g_	8.4^ab^_f_	16.4 _g_	18.5_g_	99.4^c^_f_	147.6_g_	140.6_fg_
WF+10% coarse bran+4%psyllium	13.6^d^_f_	12.2_f_	9.9_g_	12.8^ab^_f_	18.4_g_	18.7_g_	126.8^c^_f_	158.0_g_	133.9_fg_
WF+10% coarse bran+5%psyllium	13.2^d^_f_	10.5_g_	9.3_g_	13.7^b^_f_	18.3_g_	18.7_g_	131.8^c^_f_	132.6_f_	122.7_f_

Mean values (*N* = 3) with same superscripts for any parameter do not differ significantly in a column (*P* = 0.05). Mean values with same subscripts for any parameter (i.e., extensibility, resistance to extension, or area under the curve) at 45, 90, and 135 minutes do not differ significantly in a row (*P* = 0.05).

**Table 12:** Extensograph characteristics of dough made from flour, coarse bran, and psyllium blends.

Sample code	Extensibility (cm)			Resistance to extension (cm)			Area under the curve (cm^2^)		
	45	90	135	45	90	135	45	90	135
White flour (WF)	25.9^a^_f_	24.4_g_	23.5_g_	11.9^a^_f_	15.2 _g_	15.4 _g_	213.0^a^_f_	254.6_g_	247.7_g_
WF+20% coarse bran+1% psyllium	11.3^bc^_f_	9.3_g_	8.2_g_	11.5^a^_f_	18.0 _g_	18.8 _g_	95.5^b^_f_	114.7 _f_	103.6 _f_
WF+20% coarse bran+2% psyllium	11.8^b^_f_	10.6_fg_	9.5_g_	9.9^a^_f_	16.5 _g_	18.8 _h_	88.7^b^_f_	122.1 _g_	117.3 _g_
WF+20% coarse bran+3% psyllium	11.3^b^_f_	10.5_fg_	9.9_g_	10.7^a^_f_	14.9 _g_	18.4 _h_	91.6^b^_f_	116.4 _g_	120.4 _g_
WF+20% coarse bran+4% psyllium	10.9^cd^_f_	9.3_g_	7.7_h_	13.1^a^_f_	18.1 _g_	18.7 _g_	107.1^b^_f_	114.6 _g_	110.9 _g_
WF+20% coarse bran+5% psyllium	9.9^d^_f_	8.0_g_	7.0_h_	13.3^a^_f_	17.4 _g_	18.2 _g_	96.5^b^_f_	98.5_f_	88.8_f_

Mean values (*N* = 3) with same superscripts for any parameter do not differ significantly in a column (*P* = 0.05). Mean values with same subscripts for any parameter (i.e., extensibility, resistance to extension, or area under the curve) at 45, 90, and 135 minutes do not differ significantly in a row (*P* = 0.05).

## Data Availability

All the research data is included in the manuscript, and more data is available with the client, as it was a client sponsored project.
